# The status and factors associated with the mental well-being of university students in China: a cross-sectional study

**DOI:** 10.3389/fpsyt.2026.1693367

**Published:** 2026-03-11

**Authors:** Jingjing Zhang, Lawrence T. Lam

**Affiliations:** 1 Faculty of Medicine, Macau University of Science and Technology, Macau, Macau SAR, China; 2 School of Management, Putian University, Putian, Fujian, China; 3 Faculty of Medicine and Health, The University of Sydney, Sydney, NSW, Australia; 4 Faculty of Health, University of Technology Sydney, Sydney, NSW, Australia

**Keywords:** adolescents, associated factors, China, mental well-being, prevalence, SWEMWBS

## Abstract

**Objective:**

While increasing attention has been given to the mental health challenges faced by university students, there remains a lack of research specifically focused on their positive mental well-being, particularly in the East Asia region. To address this gap, the current study investigates the current status and factors associated with the mental well-being of young people in Fujian, China.

**Methods and materials:**

This cross-sectional survey was conducted among students from seven universities in Fujian Province, China. Participants’ mental well-being was assessed using the Short Warwick-Edinburgh Mental Well-being Scale (SWEMWBS). Coping strategies were measured using the Coping Inventory for Stressful Situations (CISS), and resilience was assessed using the two-item Connor-Davidson Resilience Scale (CD-RISC2). Hierarchical regression analyses were performed to examine the associations between mental well-being and various personal, familial, health-related, behavioral, and psychosocial variables.

**Results:**

Nearly one-third of respondents could be categorized as having a low level of mental well-being. After adjusting for covariates, task-oriented coping showed a positive association with mental well-being, while avoidance-oriented coping was negatively associated. Resilience was also positively related to mental well-being. Additionally, better sleep quality and longer sleep duration were positively associated with mental well-being, whereas self-harm behavior was negatively associated. Regular exercise was positively associated with mental well-being.

**Conclusions:**

Health behaviors such as regular exercise, better sleep quality and sufficient sleep, as well as psychosocial factors including resilience and positive coping, are identified as important for good mental well-being among university students in China.

## Introduction

Mental ill health among young people has long been recognized by the World Health Organization (WHO) as a global public health issue with one in seven 10-19-year-olds experiences a mental health disorder, and over one billion people worldwide currently live with such a condition (1). Mental health problems in university students are prevalent and studies indicated a higher burden than other adolescents. A recent meta-analysis involving 100,187 students found that the pooled prevalence of depression and anxiety symptoms among college students was 33.6% and 39.0%, respectively (2). Another global umbrella review including 8,706,185 participants, found high pooled prevalence of mental disorder symptoms mild depression (35.41%), mild anxiety (40.21%), and stress (36.34%) among university students worldwide (3). Similar results were also reported in China with prevalence estimates of 28.4% for depressive symptoms and 25.1% for anxiety symptoms among university students obtained from two different meta-analytical studies (4, 5). The 2024 National Survey on the Mental Health Status of University Students revealed that around 19% of students were at risk of mild-to-severe depression, with first- and second-year students exhibiting significantly higher levels of risk (6). These findings suggest that mental health problems in university students are a significant issue that require attention.

Importantly, mental health encompasses both negative and positive dimensions. Mental disorders are reflected in signs, symptoms, and impairment, whereas mental well-being represents positive functioning and optimal psychological experience (7, 8). Although mental distress is typically associated with lower well-being, the absence of a mental disorder does not necessarily imply high mental well-being (7, 8). Therefore, focusing solely on mental illness may overlook students who are not clinically symptomatic but still experience low well-being.

The World Health Organization (WHO) advocates that mental well-being focuses on positive aspects of mental health and closely related to positive psychology (9). It refers to a state in which individuals are able to function effectively, develop positive relationships, and realize their potential across different domains of life. Consistent with the mental health continuum perspective, mental well-being is increasingly recognized as a related but distinct construct from mental ill-health (7, 8). In recent years, different instruments have been designed to measure mental well-being. For instance, the Mental Health Continuum–Short Form (MHC-SF) captures emotional, psychological, and social well-being (10) while the Warwick–Edinburgh Mental Well-being Scale (WEMWBS) focuses on positive mental functioning and affective experience (11). These tools reflect the integration of hedonic aspects—such as happiness and life satisfaction—and eudaimonic aspects—such as self-realization and positive functioning (8).

For the assessment of mental well-being, WEMWBS and its short form (SWEMWBS) demonstrate strong psychometric properties and have been validated across a wide range of languages and cultural contexts, making them suitable for population-level monitoring (11, 12). In East Asia, Chinese and Korean versions of WEMWBS/SWEMWBS have been validated and applied in samples from Hong Kong, Macau, and South Korea (13–15), including recent IRT-based validation evidence in Chinese adult populations (16). Longitudinal evidence also suggests that higher mental well-being (e.g., flourishing) is prospectively associated with a lower risk of incident mood and anxiety disorders (17).

In terms of the theoretical framework for the studies in mental well-being, particularly significant correlating factors, a well-established model is yet to be developed. However, since mental well-being is strongly related to health behavior, an appropriate health behavioral model could be a good analogue for studying mental well-being. Problem Behavior Theory (PBT), developed by Jessor, proposes that youth development is shaped by the interaction of three major systems: the personality system, the perceived environment system, and the behavior system (18). Although originally formulated to explain adolescent problem behaviors, PBT has been extended to behavioral health research, highlighting the embeddedness of behavioral practices within a comprehensive explanatory network of psychosocial dispositions and social-environmental influences (19). As such, this theory is adopted as the working model of the current study in determining important study factors. Accordingly, the present study examines potential correlates of mental well-being across four domains guided by PBT: (i) personal factors (discipline, university grade, and gender), (ii) familial factors (family structure, single-child status, left-behind experience, and parental education), (iii) health-related and behavioral factors (exercise, hospital experience, sleep duration, sleep quality, and self-harm), and (iv) psychosocial factors (resilience and coping strategies). These domains were selected based on the multi-system perspective of PBT to organize and examine potential correlates of mental well-being across personal, familial, health-related, behavioral, and psychosocial.

Despite the broader international research in mental well-being using strongly validated instruments, such as WEMWBS and SWEMWBS, large-scale, theory-informed studies in mainland China university student populations remain relatively limited (20). Most existing studies have focused on translation and psychometric validation, with only a few applying the scales in specific groups such as healthcare workers (20, 21). To the authors ‘knowledge, evidence in university student samples remains limited beyond psychometric validation, and large-scale, theory-informed studies using a simplified-Chinese version of the WEMWBS/SWEMWBS appear to be scarce. Consequently, little is known about mental well-being related important personal, familial, health behaviors, and other psychosocial factors in this population, which constitutes 3.4% of the total population in China (22, 23).

This study aims to fill the knowledge gap by assessing the current status of mental well-being among university students in Fujian Province, China. As an exploratory study, it also aims to investigate the basic association between the personal, familial, health-related, behavioral, and psychosocial factors and mental well-being in this population. It is hypothesized that these factors are independently associated with mental well-being, either positively or negatively, depending on the nature of the factors. Given the cross-sectional design, the study focuses on associations between potential factors and mental well-being, rather than causal or mediational pathways, which require longitudinal or experimental data to establish temporality (24, 25).

## Methods

### Study design and participants

This study was a population-based cross-sectional health survey utilizing a self-administered online questionnaire for data collection. The survey was conducted between April 28 and May 28, 2025, and targeted full-time undergraduate students aged 18 to 24 years enrolled at seven universities in Fujian Province, China. In China, undergraduate programs typically last four years, and the age range of 18–24 years aligns with the usual enrolment and completion age of full-time university students. Eligible participants met the following criteria: 1) Aged between 18 and 24 years; 2) Enrolled as a full-time undergraduate student at one of the seven participating universities in Fujian Province during the study period; and 3) Able to read and comprehend Chinese, as the survey questionnaire was administered in Chinese. Students of any ethnicity who met this requirement were eligible for inclusion. Students who were on leave of absence, exchange students residing outside Fujian Province during the survey period, or those unable to comprehend written Chinese were excluded from the study.

Participants were recruited through collaboration with the administrative offices and student affairs departments of each participating university. The university management assisted in disseminating the online survey invitation to all eligible students via official internal communication channels, including student email lists, online campus platforms, and social media groups. Students were encouraged to participate in the survey through public notices and direct email invitations. The total number of potential participants exceeded 130,000, accounting for 11.4% of the undergraduate population in Fujian Province. This broad sampling frame included young people of the whole province that could provide a sample with sufficient representativeness. Ethics approval was granted by the Faculty Ethics Research Committee of the Faculty of Medicine, Macau University of Science and Technology (MUST-FMD-3240004069).

### Outcome measure and the potentially related factors

The aim of the study was to examine the current status of mental well-being and the related personal, familial, behavioral, and psychosocial factors of young people in China. These variables were measured with validated instruments or methods described below.

### Mental well-being

Participants’ mental well-being was measured using the Short Warwick–Edinburgh Mental Well-being Scale (SWEMWBS) (26). The original Warwick–Edinburgh Mental Well-being Scale (WEMWBS), developed in the UK, has demonstrated strong psychometric properties, with high internal consistency (Cronbach’s α = 0.89), and one-week test–retest reliability (r = 0.83), and unidimensional construct validity across different populations and languages (12). The short version (SWEMWBS) was later developed and validated using Rasch modeling, showing good fit and interval-level measurement (26). In Hong Kong, Sun et al. (13) validated the SWEMWBS in the general population, while Fung (20) validated both the WEMWBS and SWEMWBS among university students in mainland China. Further validation was conducted in a population-based sample in Macau using both classical test theory and item response theory, confirming its unidimensionality and satisfactory measurement invariance across gender (14, 16). These studies support the use of SWEMWBS for assessing mental well-being among Chinese youth. The internal consistency Cronbach’s alpha of the scale in this study sample was 0.96. The short form of the Warwick–Edinburgh Mental Well-being Scale (SWEMWBS) allows classification of raw scores into three levels of mental well-being: low (scores 7–19), moderate (scores 20–27), and high (scores 28–35), as recommended in the official user guide (12).

### Personal factors

Demographic variables included gender (male/female), grade level (Year 1 to Year 4), academic discipline (e.g., Business, Humanities, Natural and applied sciences, and Social sciences), and only-child status (yes/no). These variables were self-reported by participants through the questionnaire. In addition, participants were asked whether they had experienced being left behind during childhood (yes/no).

### Familial factors

Family structure was categorized as either a conventional family (living with both biological parents) or others (e.g., single-parent or grandparent-led households). Parental education levels were reported separately for the mother and father, and classified as university or above, secondary education, or below secondary education.

### Health-related factors

Participants were asked to report on their physical activity over the past week, with predefined response options indicating the frequency of exercise. Responses were classified into two categories: regular exercise (3–7 times per week) and less regular or no exercise (0–2 times per week). Hospitalization history was assessed via a single self-reported item inquiring whether the participant had been hospitalized due to illness or injury within the past three months. The three-month timeframe was selected because it is commonly used in population health surveys as a recent health indicator and helps minimize recall bias.

### Sleep quality

Sleep quality was evaluated using the 2-item Pittsburgh Sleep Quality Index (PSQI-2), a brief screening tool based on the validated full PSQI (27). Participants were asked to rate their overall sleep quality and report their average sleep duration over the past month. Scores range from 0–6, with higher scores indicating poorer sleep quality. For primary analyses, we dichotomized sleep quality at ≥ 2 (indicative of poor sleep quality) following validation evidence (28**).** For descriptive purposes, we also report study-defined categories: 0–2 (good sleep quality), 3–4 (possible sleep problems), and 5–6 (poor sleep quality). Prior validation has shown acceptable psychometric performance for the PSQI-2; for example, de Menezes-Júnior et al. reported excellent internal consistency (α = 0.94; ω = 0.85) and, using full PSQI > 5 as the reference, sensitivity of 77.9% and specificity of 73.8% for the ≥ 2 cut-off (28). While it does not capture all dimensions of sleep assessed by the full PSQI, the PSQI-2 is considered a valid short screening tool for identifying individuals at risk of poor sleep quality in the context of large-scale or time-limited research (28). The reliability of the 2-item scale in this study sample was checked yielding a Cronbach’s alpha value of 0.70.

### Sleep duration

Participants were asked to report their average sleep time in hours per night. Reported sleep durations were then classified, informed by the National Sleep Foundation’s recommendations, into three groups: short sleep (<6 hours), normal sleep (6–9 hours), and long sleep (>9 hours). This classification reflects established public health guidelines, where 7–9 hours of sleep is considered optimal for adults, and sleep far outside the NSF-recommended range is generally not recommended and may compromise health and well-being. Values of exactly 6 or 9 hours were included in the normal sleep category, consistent with established sleep duration guidelines (29).

### Behavioral factors

#### Self-harm behaviors

Self-harm was assessed using a multiple-choice item asking respondents whether they had engaged in any self-injurious behaviors in the past three months. The question included several specific behaviors, such as cutting, burning, or hitting oneself. Students who selected any of these behaviors were recoded as “Yes”, otherwise were considered as “No”.

### Psychosocial factors

#### Coping Styles

Coping styles were assessed using the Coping Inventory for Stressful Situations (CISS) short form (30). The CISS is a validated tool that evaluates different coping strategies in response to stress, including task-oriented, emotion-oriented, and avoidance-oriented coping (including distraction and social diversion) (30, 31). Each item is rated on a Likert scale, and subscale scores were calculated and analyzed as continuous variables. The scale was translated into Chinese by Li et al. (31) It demonstrated acceptable reliability (Cronbach’s α = 0.66–0.81) and construct validity in Chinese university students, with good model fit (RMSEA = 0.06, CFI = 0.91, NNFI = 0.91) and test-retest reliability (r = 0.65–0.78) (31). The Cronbach’s alpha of the scale in this sample was 0.92.

#### Resilience

Psychological resilience was measured using the 2-item Connor–Davidson Resilience Scale (CD-RISC2) (32). The scale was derived from the original Connor–Davidson Resilience Scale (CD-RISC) with 2 items extracted (“Able to adapt to change” and “Tend to bounce back after illness or hardship”). This short scale showed significant correlations with the remaining 23 items of the CD-RISC with a correlation coefficient of 0.78 (p<0.001) (32). It also demonstrated good convergent validity with significant correlations with the Perceived Stress Scale (PSS) and the Sheehan Stress Vulnerability Scale (SVS) yielding a coefficient of -0.51 and -0.61 (p<0.001) respectively (32). The scale has a total score ranging from 0 to 8, with higher scores indicating greater resilience (32). In Vaishnavi et al., the mean CD-RISC2 score was 6.91 in the general population, and lower mean scores were observed in clinical groups, including depression (5.12), generalized anxiety disorder (4.96), and PTSD (4.70) (32). The reliability Cronbach’s alpha value of the 2-item scale in this study sample was 0.90. Given the large sample and the length of the survey questionnaire, the 2-item scale was chosen for its recommendation of being a good quick screening tool rather than for clinical usage, although the full scale provides a more comprehensive assessment of the construct.

### Data analysis

All statistical analyses were performed using IBM SPSS Statistics for Windows, version 29.0 (IBM Corp., Armonk, NY, USA). Descriptive statistics were used to summarize the mental well-being status and the characteristics of the participants and key study variables. Means and standard deviations were reported for continuous variables, while frequencies and percentages were used for categorical variables. Bivariate analyses were conducted to examine the unadjusted associations between the potential factors and mental well-being. For categorical factors, such as disciplines, year, and gender, group comparisons of the mean mental well-being scores were conducted using independent-samples t-tests or one-way ANOVA, depending on the number of groups. Pearson correlation coefficients were calculated to assess bivariate associations between continuous variables. A selection criterion of p < 0.20 was used for identifying variables to be included in the next phase of multiple linear regression analysis, following common recommendations in epidemiological modelling to avoid prematurely excluding potentially important predictors at the initial screening stage. To further explore the factors associated with mental well-being, multiple linear regression analyses were conducted. Variables were entered in four sequential blocks: 1) Demographic characteristics: gender, grade level, academic discipline, and single-child status; 2) Familial factors: family structure, and parental education levels; 3) Behavioral and health-related factors: sleep quality, self-harm behavior, physical activity frequency, and hospitalization history; and 4) Psychosocial factors: coping styles and psychological resilience. As an exploratory study, no interaction between study variables were considered in the regression analyses. A two-tailed p-value of less than 0.05 was used for all hypothesis testing.

## Results

A total of 12,937 students responded to the online questionnaire. After removing incomplete and invalid responses, 10,799 valid questionnaires were retained. Among these, 8,024 respondents residing in Fujian Province were included in the final analysis, resulting in an effective inclusion rate of 62.0%.

The descriptive statistics of the demographic, personal, family, behavioral, psychosocial, and outcome variables were presented in **Table 1**. The majority of participants were male (62.1%), and most students were enrolled in natural and applied sciences (55.8%), followed by humanities (20.2%), business (15.4%), and social sciences (8.6%). In terms of academic year, over 40% were Year 1 students. Most participants reported not being a single child (81.2%) and not having left-behind experience (88.0%). Family background analysis indicated that 86.8% of respondents came from conventional family structures. Regarding parental education, the majority of fathers and mothers attained at least a secondary education (68.4% and 61.2%, respectively). Health-related factors showed that 63.3% of students exercised less regularly or not at all, and 14.6% had previous hospitalization experience. Over 19.7% of students reported either short or long sleep duration, and 17.5% showed signs of sleep problems or poor sleep quality. Self-harm was reported by 4.2% of students. With respect to coping strategies, the average scores for task-oriented, emotion-oriented, and avoidance-oriented coping were 20.8 (s.d. = 4.4), 19.0 (s.d. = 5.1), and 23.4 (s.d. = 5.4), respectively. Nearly two-thirds of students (65.5%) exhibited high psychological resilience. For the primary outcome, mental well-being, the mean SWEMWBS score was 25.1 (s.d. = 5.9). Based on score distributions, 31.6% of participants could be categorized as having low level of mental well-being, 28.7% as moderate, and 39.7% as high.

**Table 1:** Descriptive statistics of the familial, health-related, behavioural, psychosocial variables and mental well-being (N = 8024).

Variables	Frequency(%)/Mean (s.d.)
Demographics	
Discipline	
Business	1235(15.4%)
Humanities	1621(20.2%)
Natural and applied sciences	4481(55.8%)
Social sciences	687(8.6%)
University grade	
Year1	3330(41.5%)
Year2	2068(25.8%)
Year3	2041(25.4%)
Year4	585(7.3%)
Gender	
Male	4986(62.1%)
Female	3038(37.9%)
Familial factors	
Family structure	
Conventional family	6967(86.8%)
Others	1057(13.2%)
Single child	
Yes	1508(18.8%)
No	6516(81.2%)
Left behind experience	
Yes	961(12.0%)
No	7063(88.0%)
Father’s education	
University or above	1387(17.3%)
Secondary education	5487(68.4%)
Below secondary education	1150(14.3%)
Mother’s education	
University or above	2333(29.1%)
Secondary education	4914(61.2%)
Below secondary education	777(9.7%)
Health-related factors	
Exercise	
Less regular/no exercise	5081(63.3%)
Regular exercise	2943(36.7%)
Hospital experience	
Yes	1173(14.6%)
No	6851(85.4%)
Sleep duration#	
Normal	5864(73.1%)
Short	247(3.1%)
Long	1330(16.6%)
Mean (s.d)	8.1 (1.3)
Sleep quality	
Good sleep quality	6622(82.5%)
Possible sleep problems	1217(15.2%)
Poor sleep quality	185(2.3%)
Mean (s.d.)	1.6 (1.2)
Behavioural factors	
Self-harm	
Yes	333(4.2%)
No	7691(95.8%)
Psychosocial factors	
Resilience	
High	5255(65.5%)
Moderate	2303(28.7%)
Low	466(5.8%)
Mean (s.d.)	7.4 (1.7)
Coping strategies	
Task-oriented	20.8(4.4)
Emotion-oriented	19.0(5.1)
Avoidance-oriented	23.4(5.4)
Study outcome	
Mental wellbeing	
Low	2533(31.6%)
Medium	2308(28.7%)
High	3183(39.7%)
Mean (s.d.)	25.1(5.9)

#583 cases were excluded due to missing sleep duration data.

**Table 2** summarized the unadjusted associations between personal, familial, behavioral, psychosocial variables and mental well-being. Significant differences in mean SWEMWBS scores were observed across academic disciplines (p< 0.001), family structure (p< 0.001), and parental education levels (both p< 0.001). Students with left-behind experience or previous hospitalization had significantly lower well-being scores (p< 0.001). Health behaviors such as regular exercise, and longer sleep duration (r = 0.10, p < 0.001) were positively associated with well-being, while poor sleep quality (r= −0.26, p < 0.001) and self-harm was strongly negatively associated (p< 0.001). Among coping styles, task-oriented coping showed a strong positive correlation with well-being (r= 0.54, p< 0.001), whereas avoidance-oriented coping had a weaker positive correlation (r= 0.27, p< 0.001); emotion-oriented coping was not significantly associated (r= −0.001, p= 0.993). No significant differences were found by gender, university grade, or only-child status (p > 0.05).

**Table 2:** Unadjusted association between personal, familial, health-related, behavioural, psychosocial variables and mental well-being (N=8024).

Variables	Results of comparisons
Demographics	
Discipline	F_(3, 5757)_ = 9.78, p < 0.001
University Grade	F_(3, 5757)_ = 0.21, p = 0.893
Gender	t_(5759)_ = 0.22, p = 0.825
Familial factors	
Family structure	t_(1107.98)_ = 5.05, p < 0.001
Single child	t_(1892.99)_ = 1.81, p = 0.071
Left behind experience	t_(5759)_ = -4.13, p < 0.001
Father’s education	F_(2, 5758)_ = 13.71, p < 0.001
Mother’s education	F_(2, 5758)_ = 9.94, p < 0.001
Health-related factors	
Exercise	t_(3934.84)_ = 7.35, p < 0.001
Hospital experience	t_(1480.56)_ = -4.21, p < 0.001
Sleep Duration	r = 0.10, p < 0.001
Sleep Quality	r = -0.26, p < 0.001
Behavioural factors	
Self-harm (No)	t_(369.60)_ = 16.73, p < 0.001
Psychosocial factors	
Coping strategies	
Task-oriented	r = 0.54, p < 0.001
Emotion-oriented	r = -0.01, p = 0.993
Avoidance-oriented	r = 0.72, p < 0.001
Resilience	r = 0.57, p < 0.001

Multiple linear regression analyses revealed that several psychosocial and behavioral factors were significantly associated with mental well-being (**Table 3**). Normality checks of the outcome and other included variables were conducted and the results indicated no particular issues. Multicollinearity diagnostics indicated acceptable levels, with all variance inflation factor (VIF) values below 3.0, suggesting no serious concerns regarding multicollinearity among the included variables. Among the coping strategies, task-oriented coping showed a positive association (β=0.25, p < 0.001), while avoidance-oriented coping was negatively associated (β= −0.56, p < 0.001) after adjusting for covariates. Resilience was also positively related to mental well-being (β=1.19 p <0.001). In terms of behavioral factors, poor sleep quality (β=−0.42, p<0.001) were negatively associated with mental well-being, whereas longer sleep duration (β= 0.19, p <0.001) and regular exercise (β= 0.40, p= 0.013) were positively associated. Additionally, past hospitalization experience was found to be negatively related to mental well-being (β= −0.52, p=0.002), while students without self-harm experience showed a significant positive association (β=1.62, p<0.001).

**Table 3:** Results obtained from the multiple linear regression analyses (N=8024).

Variables in the final model	β	SE(β)	t	p	95% CI for β (Lower)	95% CI for β (Upper)	VIF
Health-related factors							
Exercise	0.40	0.15	2.47	0.013	0.08	0.66	1.02
Hospital experience	-0.52	0.17	-3.03	0.002	-0.85	-0.18	1.01
Poor sleep quality	-0.42	0.06	-6.92	<0.001	-0.54	-0.30	1.09
Sleep duration	0.19	0.05	3.52	<0.001	0.09	0.30	1.02
Behavioural factor							
Self-harm (No)	1.62	0.32	5.02	<0.001	1.00	2.25	1.07
Psychosocial factors							
Coping							
Avoidance-oriented	-0.56	0.02	-23.10	<0.001	-0.62	-0.52	2.97
Task-oriented	0.25	0.03	9.29	<0.001	0.19	0.30	2.57
Resilience	1.19	0.07	7.70	<0.001	1.07	1.32	1.30

Model statistics: F_(8, 7431)_ = 460.30, p < 0.001.

R²= 0.462.

## Discussion

This study aimed to investigate the current status and factors associated with the mental well-being of university students in the Fujian province in China. As shown in the results, nearly one-third of respondents (31.6%) could be categorized as having a low level of mental well-being. Compared with other student populations, the mean SWEMWBS score in this study (mean = 25.1, s.d. = 5.9) was slightly higher than that reported among Norwegian upper secondary students aged 15–21 with a mean of 24.9 (s.d. = 5.0) (33), and clearly higher than the mean score of 20.3 (s.d. = 3.9) reported in an international sample of further and higher education students after returning to in-person learning following Covid-19 restrictions (34). To the knowledge of the authors, this is the largest survey of mental well-being among university students in mainland China to date (N = 8,024). Unlike most existing Chinese studies that have focused on depression and anxiety, or on psychometric validation in specific populations, this study specifically examines positive mental well-being using a validated simplified-Chinese SWEMWBS in a large student sample. By adopting PBT as an organizing framework, correlates across personal, familial, health-related, behavioral, and psychosocial domains were examined simultaneously, and contextually relevant factors such as hospitalization history and self-harm experience were identified as significant correlates. Another Chinese head-to-head psychometric study using the SWEMWBS and EQ-5D-5L was conducted with a sample of 500 respondents (35).

The working model of the study is based on the theoretical or conceptual framework of Jessor’s PBT with the main study factors generated from various influential domains, such as personal, family, environmental, and psychosocial. The model suggests that there could be risk and protective factors in each of these domains that are associated with the outcome. This provides a structure for understanding the results obtained. For factors associated with mental well-being in Chinese university students, the results indicated a few significant variables. Health behaviors such as regular exercise, sufficient sleep duration, and good sleep quality were positively associated with well-being. These results were consistent to those reported in the literature. For example, in Fox et al.’s review it was reported that moderate regular exercise was associated with improvements in mental well-being (36). In terms of other health-related factors, poor sleep quality and shorter sleep duration were negatively related to mental well-being, which is also in line of the findings in the literature. In the study by Clayborne et al. it was found that good sleep quality was strongly associated with better mental well-being, with individuals reporting good sleep quality having 1.52–4.24 times higher odds of positive mental health (37). Regarding the psychosocial factors, resilience was strongly and positively associated with the mental well-being of these university students. This result was also consistent with the findings in the literature. A study of Chinese university students reported that resilience and coping styles significantly predicted subjective well-being, indicating a positive role of resilience in students’ overall subjective well-being (38).Task-oriented coping was positively associated with mental well-being, while avoidance-oriented coping was negatively related. This finding is also consistent with the phenomena presented in the literature. In a large Australian adolescent sample, Frydenberg and Lewis found that negative avoidant coping was consistently and negatively associated with well-being and positively associated with psychological distress, while active coping showed a positive correlation with well-being when coping effectiveness was not controlled (39). The results obtained from this study render further support to the PBT.

The findings of this study have several practical implications for promoting mental well-being in young people. First, the results suggest that resilience and positive coping are significant protective factors of mental well-being of young people. Second, positive health behaviors such as regular exercise, good sleep quality, and sufficient sleep are also associated with better mental well-being. These results provide insights into the important target areas in the ongoing efforts in promoting better mental well-being in young people. In the design of future mental well-being intervention programs, it is recommended that these areas should be the main foci. These intervention programs should help young people in building a positive attitude towards themselves. In addressing the protective factors, they should include protective elements in enhancing resilience and fostering positive coping. For reducing the impact of risk factors, these programs should also include contents that can promote good health behavior, such as regular physical activity and psychoeducation. These elements have also been identified through the systematic review study by Lam et al. and in other studies (40–42).In addition, particular attention should be given to students with a history of hospitalization or self-harm experience, as these were identified as significant risk factors associated with lower mental well-being in this study.

As for all studies, there are strengths and limitations. In terms of the strengths, first, it utilized a large and representative sample of university students in Fujian. This ensures better generalizability of the findings to similar populations. Second, the study used well-validated and reliable instruments for mental well-being, resilience, and health-related behaviors, to ensure robust assessments of all core variables and reducing information biases. Third, multi-influencing factors, including familial factors, health-related matters, behavioral issues, and psychosocial factors, were considered in the analysis, allowing for comprehensive understanding of the determinants of mental well-being in young people. Regarding the limitations, the study design being a cross-sectional study precludes any inference of direction of association or causality. Associations observed in this study should not be interpreted as causal. Secondly, self-reported data are subject to recall biases, social desirability biases, and individual interpretations, the potential influence of the accuracy of reported behaviors and mental well-being. Thirdly, although the sample was large and diverse, it was limited to a single province in China, regional and cultural differences cannot be generalized to other settings or populations. The culturally specific factors included in this study, such as left-behind experience and single-child status, reflect the unique sociocultural context of mainland China. However, culture was not formally tested as a moderator or mediator in this study, and future cross-cultural studies are needed to examine potential moderating effects of culture on the observed associations. Fourthly, the use of a two-item resilience scale (CD-RISC2), while psychometrically validated, may not fully capture the multidimensional nature of resilience, and future studies should consider using more comprehensive resilience measures where feasible. In the future, studies should employ longitudinal-experimental designs or experimental designs to establish causal relationships and enrich our understanding of mental well-being and its associated factors. Increasing diversity among study participants would strengthen the external validity of the findings.

## Conclusion

In summary, this cross-sectional study revealed that nearly one-third of university students in China had a lower level of mental well-being. Some health behaviors, such as regular exercise, better sleep quality and sufficient sleep, as well as psychosocial factors including resilience and positive coping are identified as important for good mental well-being. The results provide practical guidance for the development of accessible, scalable, and student-centered mental well-being promotion strategies on campus.

## Data Availability

The raw data supporting the conclusions of this article will be made available by the authors, without undue reservation.
